# Possible Roles of Specific Amino Acids in β-Tubulin Isotypes in the Growth and Maintenance of Neurons: Novel Insights From Cephalopod Mollusks

**DOI:** 10.3389/fnmol.2022.838393

**Published:** 2022-04-14

**Authors:** Richard F. Ludueña

**Affiliations:** Department of Biochemistry and Structural Biology, University of Texas Health Science Center at San Antonio, San Antonio, TX, United States

**Keywords:** β-tubulin, β-III, cephalopods, vertebrates, cysteine cluster, C-termini, cancers, C239

## Abstract

Microtubules, are formed of the protein tubulin, which is a heterodimer of α- and β-tubulin subunits. Both α- and β-tubulin exist as numerous isotypes, differing in amino acid sequence and tissue distribution. Among the vertebrate β isotypes, βIII has a very narrow distribution, being found primarily in neurons and in advanced cancers. The places in the amino acid sequence where βIII differs from the other β isotypes are highly conserved in evolution. βIII appears to be highly resistant to reactive oxygen species and it forms highly dynamic microtubules. The first property would be very useful in neurons, which have high concentrations of free radicals, and the high dynamicity would aid neurite outgrowth. The same properties make βIII useful in cancers. Examination of the amino acid sequences indicates a cysteine cluster at positions 124–129 in βIII (CXXCXC). This occurs in all βIII isotypes but not in βI, βII, or βIV. βIII also lacks the easily oxidized C239. Both features could play roles in free radical resistance. Many aggressive tumors over-express βIII. However, a recent study of breast cancer patients showed that many of them mutated their βI, βII, and βIV at particular places to change the residues to those found at the corresponding sites in βIII; these are all sites that are highly conserved in vertebrate βIII. It is possible that these residues are important, not only in the resistance to free radicals, but also in the high dynamicity of βIII. The cephalopod mollusks are well known to be highly intelligent and can remodel their own brains. Interestingly, several cephalopods contain the cysteine cluster as well as up to 7 of the 17 residues that are highly conserved in vertebrate βIII, but are not found in βI, βII, or βIV. In short, it is possible that we are looking at a case of convergent evolution, that a βIII-like isotype may be required for neuronal growth and function and that a structure-function study of the particular residues conserved between vertebrate βIII and cephalopod tubulin isotypes could greatly increase our understanding of the role of the various tubulin isotypes in neuronal growth and function and could aid in the development of novel anti-tumor drugs.

## Introduction

Tubulin, the subunit protein of microtubules, is an α/β heterodimer; both α- and β-tubulin exist as multiple isotypes. Tubulin isotype families appear to be phylum-specific; in other words, comparison of the amino acid sequences of the various tubulins suggests that in any given phylum, the different β-tubulin isotypes evolved from one ancestral β-tubulin and similarly for the α-tubulins (Ludueña, 2013). I shall focus here on the isotypes of vertebrate β-tubulin, in part because they have been better studied, and also because some very important anti-tumor drugs, such as the taxanes and the *Vinca* alkaloids, have binding sites that include β-tubulin (Snyder et al., 2001; Prota et al., 2014). The sequence differences among the β isotypes have been strongly conserved in evolution, suggesting that they are functionally significant (Ludueña, 2013). I shall focus mainly on the βIII isotype, which plays a major role in neurons and is required for neuronal growth and regeneration (Moskowitz et al., 1993; Moskowitz and Oblinger, 1995; Roskams et al., 1998; Latremoliere et al., 2018). The specific regions in βIII that are important for its role in neurons are not known. However, this article assembles insights from *in vitro* studies of purified αβIII, together with sequences from β-tubulins found in cancers and, surprisingly, from cephalopod mollusks and other invertebrates, to tentatively identify specific regions in βIII that may allow it to play special roles in neurons. Other vertebrate tubulin isotypes, specifically βV and βVI, will be discussed for purposes of comparison. Finally, I shall also propose experiments to test these hypotheses.

## Methods

Amino acid sequences of β-tubulins from human subjects were collected from a previously published work, which was fully approved by a local ethics panel at the Alberta Cancer Research Biobank (Wang et al., 2017). Sequences of other vertebrate and invertebrate tubulins were collected from publicly available databases and are listed individually in the tables. No animals or humans were used directly in this study. Sequences were compared to look for resemblances to each other. Tubulin is a highly conserved protein, so no particular software was required to align the sequences up through position 424, after which the highly variable C-terminal sequence begins, which will be discussed later. An examination of a large number of insect β-tubulin sequences (Nielsen et al., 2010) revealed three exceptions to this. One was *Drosophila melanogaster* β-tubulin at 60D, isoform B (NP_001286835). In this case a quick comparison of its sequence to those of other β-tubulins indicates that 5 residues have been inserted in the region of 55-60. The other exceptions were the β-tubulins from the parasitic wasp *Nasonia vitripennis* (XP_003427519) and the domestic silk moth *Bombyx mori* (NP_001036888), where 6 residues were inserted in the same region. Once these are accounted for, alignment with other β-tubulins appears to be perfect.

## Results

### Specific Properties of βIII-Tubulin

Unlike the βI and βIV isotypes that are widespread among many tissues, βIII is only abundant in nerves, but only in neurons themselves, not in glial and other supportive cells (Burgoyne et al., 1988). βIII is also found in the testis and, in lower amounts in the colon, but is very rare in other tissues (Lewis and Cowan, 1988; Roach et al., 1998). In the mammalian brain, βIII accounts for 25% of total brain β-tubulin in the mammals where it has been measured and has also been observed in the brains of chickens and fish (Ludueña et al., 1982; Detrich et al., 1987; Modig et al., 1999).

We have used isotype-specific monoclonal antibodies to purify αβII, αβIII, and αβIV from bovine brains by immunodepletion chromatography (Banerjee et al., 1992). This has allowed us to study the properties of the purified dimers *in vitro*. In this type of procedure, purification can never be perfect, so small amounts of heterologous tubulin dimers were present in the highly, but not fully, purified dimers (Banerjee et al., 1992). Nevertheless, we and others have found that αβIII has five properties that distinguish it from the other αβ dimers. First, αβIII has a much more rigid conformation than either αβII or αβIV; this was shown using a variety of approaches (Banerjee et al., 1994, 1997; Sharma and Ludueña, 1994; Schwarz et al., 1998). Second, αβIII binds with weaker affinity to the drugs vinblastine, colchicine and nocodazole, and microtubules made of αβIII are less affected by paclitaxel than are those made from αβII or αβIV (Banerjee and Ludueña, 1992; Derry et al., 1997; Xu et al., 2002; Khan and Ludueña, 2003). Third, microtubules made from almost pure αβIII have much higher dynamicity than those made from almost pure αβII or αβIV (Panda et al., 1994; Vemu et al., 2016). Interestingly, microtubules made from recombinant αβIII, which we can assume is completely free from other tubulin isotypes, exhibit very low dynamicity, which increases greatly when even small amounts of other tubulin isotypes are added (Vemu et al., 2016). Since virtually no cell expresses only a single isotype of tubulin, one can probably assume that cellular microtubules rich in βIII are highly dynamic. Fourth, βIII responds to oxidative stress by binding to glutathione, much more so than do either βI or βII (Guo et al., 2018). Finally, different approaches have shown that the presence of βIII protects the cell from oxidative or other stresses (Gan et al., 2007; Guo et al., 2010). Table 1 lists 25 positions where βIII differs from most of the other β isotypes and which are highly conserved in vertebrate evolution. I shall be focusing on a subset of these. I examined the sequences of a variety of vertebrate βIII-tubulins to look for conserved residues. The conserved and nearly conserved residues from some of these vertebrates are shown in Table 1. I did a further analysis (not shown) that included βIII-tubulins from a few examples of each class of vertebrates and of different orders or clades within each class. Altogether, the vertebrates whose βIII-tubulin sequences were examined are classified as follows.

**TABLE 1:** Unique, Conserved Positions in βIII and their locations in the 3D Structure of Tubulin*^a.b^*.

	βIII						Human							3D Position	Chemo
#	*H.s.*	*M.m.*	*G.g.*	*G.j*	*X.l.*	*S.s.*	βI	βIIA	βIIB	βIVA	βIVB	βV	βVI		
**33**	**S**	S	S	S	C	T	**T**	T	T	L	T	A	A	H1/S2 Loop	t
**35**	**N**	N	N	N	N	N	**T**	S	T	T	T	G	S	H1/S2 Loop	t
37	V	V	V	V	I	E	H	H	H	H	H	V*	R	H1/S2 Loop	
**48**	**S**	S	S	S	S	S	S	**V**	N	N	N	N	S*	H1/S2 Loop	n
55	S	S	S	S	S	S	T	A	T	T	T	S*	Y	H1/S2 Loop	
56	S	S	S	S	S	S	G	G	G	G	G	S*	G	H1/S2 Loop	
57	H	H	H	H	L	S	G	N	N	G	G	Q	R	H1/S2 Loop	
80	A	A	A	A	A	T	P	P	P	P	P	P	K	H2/S3 Loop	
83	H	H	H	H	H	Q	Q	Q	Q	Q	Q	Q	A	H2/S3 Loop	
84	L	L	L	L	L	L	I	I	I	I	I	I	L*	H2/S3 Loop	
91	I	I	I	I	I	I	V	V	V	V	V	I*	V	β-Sheet S3	
**124**	**C**	C	C	C	C	C	A	S	S	A	**A**	C*	S	α-Helix H3	t
**126**	**N**	N	N	N	N	N	S	S	S	S	**S**	H	S	H3/S4 Loop	t
**155**	**V**	V	V	V	V	I	I	**I**	I	I	I	I	I	α-Helix H4	n,t
**189**	**I**	I	I	I	I	I	V	V	V	V	**V**	V	I*	α-Helix H5	t
**218**	**A**	A	A	A	A	P	T	T	T	T	**T**	T	T	H6/H7 Loop	t
**239**	**S**	S	S	S	S	S	**C**	C	C	C	**C**	S*	S*	H7/H8 Loop	t
**275**	**A**	A	A	A	A	A	S	S	S	S	**S**	S	A	S7/H9 Loop	n
**315**	**T**	T	T	A	T	M	A	A	A	A	**A**	T*	C	β-Sheet S8	n
**332**	**A**	A	A	A	A	A	N	N	N	S	**V**	A*	S	α-Helix H10	n,d
**333**	**I**	I	I	I	I	I	V	V	V	V	**Q**	I*	V	α-Helix H10	n,d
**335**	**S**	S	S	S	S	S	N	N	N	S	**K**	S*	T	α-Helix H10	n,d
**351**	**V**	V	V	V	V	V	**T**	T	T	T	**T**	V*	V*	β-Sheet S9	n,d
**364**	**S**	S	S	S	S	S	**A**	S	S	A	P	A	A	β-Sheet S10	
**365**	**S**	S	S	S	S	A	**V**	A	A	A	**A**	S*	A	β-Sheet S10	n,d

Positions and residues in **BOLD** indicate sites of mutations in breast cancer patients where βI, βIIA or βIVB are mutated to positions corresponding to positions in βIII (also shown in **BOLD**). Note: the C-terminal amino acids (431-) were not part of this study. Positions in βV and βVI marked with an asterisk (*) are those where βV or βVI are identical with βIII and differ from the other β isotypes. Locations in the 3D structure are based on the model of Nogales et al. (1998). H = α-Helix; S = β-Sheet. For those sets of patients who had mutations, their chemotherapy (Chemo) is indicated on the far right (n = no chemotherapy; t = taxane, d = doxorubicin).

*^a^Abbreviations: H.s. = Homo sapiens (human); M.m. = Mus musculus (mouse); G.j. = Gallus gallus (chicken); G.j. = Gekko japonicus (Schlegel’s Japanese gecko); X.l. = Xenopus laevis (African clawed frog); S.s. = Salmo salar (Atlantic salmon).*

*^b^Accession numbers: Human βIII (AAL28094), Mouse βIII (NP_075768), Chicken βIII (NP_001383082), Gekko japonicus βIII (AFD53918), Xenopus βIII (NP_001088455), Salmon βIII (XP_013982514), Human βI (BAE78618), Human βIIA (NP_001060), Human βIIB (NP_821080), Human βIVA (NP_001276058), Human βIVB (AAN87335), Human βV (Q9BUF5).*

#### Mammals

Order Primates: *Homo sapiens* (human) (AAL28094); Order Rodentia: *Mus musculus* (mouse) (NP_075768); Order Artiodactyla: *Bos taurus* (cow) (NP_001070595).

#### Birds

Order Galliformes; *Gallus gallus* (chicken) (NP_001383082); Order Strigiformes: *Tyto alba* (barn owl) (XP_009969972); Order Anseriformes: *Anas platyrhynchos* (Pekin duck) (XP_005010451); Order Passseriformes: *Catharus ustulatus* (Swainson’s thrush) (XP_032925992).

#### Reptiles

Order Squamata: *Gekko japonicas* (Schlegel’s Japanese gecko) (AFD53918); Order Testudines: *Chelonia mydas* (green sea turtle) (XP_037733910).

#### Amphibians

Order Anura: *Xenopus laevis* (African clawed frog) (NP_001088455); *Bufo bufo* (common toad) (XP_040266023); Order Gymnophiona: *Geotrypetes seraphini* (Gaboon caecilian) (XP_033798150).

#### Teleosts

(Bony fish) Clade Actinopterygii, Order Gadiformes: *Gadus morhua* (Atlantic cod) (AAD56400); Clade Actinopterygii, Order Characiformes: *Astyanax mexicanus* (Mexican tetra) (XP_007244083); Clade Actinopterygii, Order Siluriformes: *Tachysurus fulvidraco* (yellowhead catfish) (XP_027015187), *Ictalurus punctatus* (channel catfish) (AHH42382); Clade Actinopterygii, Order Salmoniformes: *Salmo salar* (Atlantic salmon) (XP_013982514); Clade Crossopterygii, Order Coelacanthiformes: *Latimeria chalumnae* (coelacanth) (XP_005999257).

#### Chondrichthyes

Order Orectolbiformes: *Chyloscillium plagiosum* (White-spotted bamboo shark) (XP_043562712); Order Lamniformes: *Carcharodon carcharias* (great white shark) (XP_041047906); Order Rajiformes: *Amblyraja radiata* (thorny skate) (XP_032891568).

The residues that were conserved in every one of these βIII-tubulins and were different from those in βI, βIIA, βIIB, βIVA, βIVB, and βVIII were N35, S56, L84, C124, I189, S239, A275, and V351, suggesting that these residues are the most likely to play critical roles in the specific functions of βIII-tubulin. Table 1 shows other residues that are highly but not entirely conserved in βIII, suggesting that they too may play a role in mediating the function of βIII.

## Structure–Function Correlations in βIII

Can any of these unique properties of βIII be correlated with particular portions of the amino acid sequence of βIII?

Probably the most obvious factor is that βIII has a serine at position 239; this is true for the βIII, βV and βVI isotypes of every known vertebrate. The significance of βV and βVI will be discussed later. In contrast, every example and sub-type of βI, βII and βIV has C239 (Ludueña, 2013). In addition, virtually every animal β-tubulin has C239; the highly significant exceptions will be discussed later. Finally, most plants and most protists also have C239. Fungi, however, generally have S239.

This serine at position 239 in βIII is very likely to protect microtubules made from αβIII from oxidative stress. Many experiments have shown that C239 reacts very easily with alkylating agents resulting in inhibition of microtubule assembly (Little and Ludueña, 1985; Bai et al., 1989; Yang et al., 2019). It is reasonable to propose, therefore, that the role of S239 in βIII is to prevent this effect and to protect microtubule assembly from oxidative stress. This could be adaptive for microtubule assembly in neurons, the testis and cancer cells, all of which are rich in free radicals (reviewed in Ludueña, 2013).

Another region of probable significance is the cysteine “cluster” of C124, C127, and C129. C127 and C129 are very widely conserved among eukaryotic β-tubulins; in vertebrates, however, C124 is found in all βIII and βV isotypes and in a few βVI isotypes. A cysteine cluster of similar topography (CXXCXC) is found in Von Willebrand’s factor and is involved in a sulfhydryl-disulfide interchange that accompanies polymerization of this protein (Mayadas and Wagner, 1992; Dong et al., 1994; Katsumi et al., 2000). βIII could conceivably have an intra-chain disulfide (Chaudhuri et al., 2001) and it is reasonable to speculate that this sulfhydryl cluster could react with glutathione and perhaps play a role in resistance to oxidizing agents; it could also be involved in a sulfhydryl-disulfide interchange accompanying microtubule assembly (Britto et al., 2005).

Table 1 shows the positions of the residues of interest in the secondary structure of the tubulin dimer, assigning them to α-helices or β-sheets according to the analysis of Nogales et al. (1998); based on their model, we can also speculate as to the positions of these residues in the overall structure of the tubulin dimer and the microtubule. We can state that residues 33, 35, 37, 48, 55, 56, and 57 are on the inner surface of the microtubule and that residues 80, 83, 84, 91, 124, and 126 are in regions that are involved in lateral interactions between protofilaments in the microtubule. However, none of the residues in Table 1 are specifically known to be involved in any kind of intra- or inter-dimer interaction. The closest to an exception would be residue 218, right next to T219, which is directly involved in an inter-dimer longitudinal interaction (Nogales et al., 1999).

It is not immediately obvious which amino acids are involved in the increased dynamicity of microtubules containing αβIII. This is because, even if a given amino acid is not involved directly in lateral or longitudinal interactions between tubulin dimers in a microtubule, it could play an important role in strengthening or weakening the conformational state of a tubulin molecule and thus indirectly affect microtubule dynamics as well as affecting the interactions of tubulin and microtubules with other molecules.

## Insights From Cancer Cells

Cancer cells could provide a clue. Increased dynamicity of microtubules would be adaptive for cancer cells, which have to construct mitotic spindles at higher than normal rates and also change their shapes and migrate more than do most other cells (Yamaguchi et al., 2005; Garcin and Straube, 2019; Mierke, 2019). It is not surprising, therefore, that so many cancers, especially the more advanced and metastatic ones, express large amounts of βIII (Katsetos et al., 2003). In addition, cancer cells are rich in reactive oxygen species, including free radicals, and the presence of S239 in βIII may protect microtubule assembly (Punnonen et al., 1994; Ray et al., 2000; Brown and Bicknell, 2001; Raspaglio et al., 2008). A recent study of breast cancer patients has provided some insight into this (Wang et al., 2017). If it is adaptive for a cancer cell to over-express βIII, then it might also be adaptive for the cell to mutate some of its other isotypes to become more like βIII, particularly at those positions that determine the unique properties of βIII. From the 90 patients in the study, 1167 β-tubulin isotype clones were sequenced. Many of these had mutations in βI, βIIA, or βIVB, in which residues at certain positions were mutated to the corresponding residues in βIII (Table 1; Wang et al., 2017). It is important to note that this study did not include the C-terminal region of the β isotypes. We shall return to this region later. In this study, we only examined mutations in the 25 positions that were highly conserved in the evolution of βIII and which differ from many of the other β isotypes. Of these, 15 were among the positions where a β isotype mutated to acquire the corresponding residue at that position in βIII. Mutations of A124, S126, I155, V189, T218, C239, S275, A315, N332, V333, N335, T351, and A365 to the corresponding βIII residues were correlated with cancers of high grade. In contrast, mutations at C239 to other than S239 were all correlated with low-grade cancers (Wang et al., 2017). This raises the possibility that the key to the high dynamicity of βIII may lie in that one subset of residues, specifically: C124, N126, V155, I189, A218, S239, A275, T315, A332, I333, V351, and S365. Table 1 indicates the positions where βIII differs from the β isotypes in the first category, emphasizing the strong conservation in vertebrate evolution. The table also indicates the residues that were mutated in the cancer patients. It is interesting that of the eight positions identified as being virtually universally conserved in βIII, six of these (35, 124, 189, 239, 275, and 351) are sites where one of the β-tubulins in breast cancer patients mutated to acquire the residue that βIII has in that positions.

One might ask if these mutations arose as a result of resistance to the taxanes or doxorubicin used to treat some of these patients. As shown in Table 1, the results are quite complex. Some of the patients in Table 1 had no chemotherapy; others were treated with taxanes or doxorubicin. Taxanes bind to tubulin whereas doxorubicin does not; both stress the cell, and increases in βIII expression are associated with resistance to these reagents (Gan et al., 2007). Analysis of the taxane binding site indicates that none of the residues shown in Table 1 interacts directly with taxanes (Li et al., 2000). Since taxanes alter tubulin conformation (Mitra and Sept, 2008; Yajima et al., 2012), it is possible that those mutations observed only in the patients treated with taxanes blocked the taxane-induced conformational change.

## Lessons From the βV and βVI Isotypes

The βV isotype is unquestionably the least understood of the vertebrate β-tubulin isotypes, probably because it has never been purified or prepared in a functional state. Nothing is known of its drug-binding properties, nor its dynamicity, nor its post-translational modifications, which are a major feature of all the other α- and β-tubulin isotypes (Ludueña, 2013). Nevertheless, given its sequence similarity to βIII, one can hypothesize that its functional properties may resemble those of βIII. It is therefore not surprising that βV is elevated in some tumors (Verdier-Pinard et al., 2005; Hiser et al., 2006). In fact, one can make the case that tumors can over-express βIII or βV, but usually not simultaneously, suggesting that the two isotypes play similar roles in tumor progression. Furthermore, βV is briefly expressed in the neurons of newborn mice, suggesting that it may play a role in generation of neurons (Guo et al., 2011). Table 1 shows that several residues in βIII that I have speculated may be associated with high dynamicity are also present in βV; it is reasonable to speculate that βV creates dynamic microtubules that could be required for neurogenesis in these mice. This is supported by the finding that βV, when incorporated into cellular microtubules *in vitro*, causes them to break down (Bhattacharya and Cabral, 2004; Salinas et al., 2014). Also, βV has an almost permanent presence in the lungs and nasal tissues, which are exposed to oxygen in the air we breathe (Nakamura et al., 2004). The nasal passages produce the free radical NOź (Chatkin et al., 1999), which probably inhibits microtubule assembly (Landino et al., 2007), so having microtubules enriched in βV may be adaptive.

To summarize, if the arguments made above about structure-function correlations are correct, they could also apply to βV. Since βV has S239 instead of C239, its assembly is likely to not be inhibited by free radicals such as superoxide anion or nitric oxide. Since βV has the cysteine “cluster” (C124, C127, C129) it is likely to interact with glutathione; this feature may also protect against reactive oxygen species. Table 1 shows the 12 positions in βV where it is the same as βIII but different from the other β isotypes. Again, the C-termini are not included. Of these 12 positions, 8 are at key positions where mutations in the βI, βIIA, or βIVB isotypes are associated with more aggressive cancers (Wang et al., 2017). If these residues are involved in the higher dynamicity of βIII, then it is likely that βV also has high dynamicity, which would be consistent with its expression in cancer cells.

The vertebrate βVI isotype is found in the microtubules of platelets and erythrocytes (Schwer et al., 2001); the exception is mammals, whose erythrocytes do not contain microtubules (Behnke, 1970a), although βVI is found in mammalian platelets. Microtubules of these cells exhibit dynamic behavior, but of a different kind than that of other types of microtubules; their dynamicity appears to be manifested by rapid sliding, coiling and bundling rather than by rapid assembly and disassembly (Italiano et al., 1999; Patel et al., 2005; Van Dijk et al., 2018). In *Xenopus*, erythrocyte microtubules contain βVI and they are not at all dynamic (Gambino et al., 1985). In fact, unlike the case with βIII, the presence of βVI in cellular microtubules has been shown to suppress microtubule dynamics (Yang et al., 2011). βVI has some minor resemblance to βIII and βV in that it has S239. However, mammalian βVI does not appear to have the “cysteine cluster” (CXXCXC). Table 2 shows that, of the 15 unique conserved positions where βIII differs from the βI, βIIA, βIIB, βIVA, and βIVB isotypes, human βVI only resembles βIII at five positions, of which four are positions where the other β isotypes mutated to resemble βIII in the breast cancer study. In other words, we cannot make a good case that βVI is likely to contribute to increased dynamicity of microtubules that contain it. Interestingly, there is an exception to this. βVI from birds and some reptiles contains S239 together with the full cysteine cluster at positions 124–129. In contrast to the five positions where human βVI has the same residues as the conserved positions in βIII, chicken βVI has the same residues as βIII at 10 positions, of which six are at the positions where the βI, βIIA, βIIB, βIVA, and βIVB isotypes, mutated to resemble βIII.

**TABLE 2:** Key Positions in Vertebrate βVI-Tubulin Isotypes*^a^*.

											Human	
Position	Human	Mouse	Chicken	Duck	Gecko	Turtle	*Xenopus*	*Channa*	Molly	Cod	βIII	βV
33	A	A	A	A	G	T	Q	T	T	T	S	A
35	S	S	N	N	N	N	S	F	L	N	N	G
37	R	C	C	R	C	H	H	E	E	V	V	V
48	S	S	N	N	N	N	N	N	N	N	S	N
55	Y	Y	Y	Y	Y	H	H	H	H	S	S	S
56	G	G	S	S	S	S	S	G	G	S	S	S
57	R	K	H	H	H	K	H	G	G	N	H	Q
80	K	R	K	K	K	K	S	R	R	T	A	P
83	A	V	P	P	P	P	P	A	A	Q	H	Q
84	L	L	L	L	L	L	L	L	L	L	L	I
91	V	V	I	I	I	I	I	I	I	I	I	I
124	S	S	C	C	C	C	S	S	S	C	C	C
126	S	S	S	S	C	S	S	S	S	N	N	H
155	I	I	I	I	I	I	I	I	I	I	V	I
189	I	I	I	I	I	I	I	V	V	V	I	V
218	T	T	T	T	P	R	T	T	T	T	A	T
239	S	S	S	S	S	S	S	S	S	S	S	S
275	A	A	A	A	A	A	S	A	P	A	A	S
315	C	C	C	C	C	C	G	G	G	T	T	T
332	S	S	S	A	S	A	S	A	A	N	A	A
333	V	I	V	V	I	I	V	I	M	V	I	I
335	T	T	T	T	T	T	S	Q	Q	N	S	S
351	V	V	V	V	V	V	V	V	V	V	V	V
364	A	A	A	A	S	A	A	A	A	S	S	A
365	A	A	A	A	A	A	S	S	S	A	S	S

*The table shows the relative conservation of the sequences of βVI-tubulin in vertebrates (bony fish, amphibians, reptiles, birds and mammals). In this table only the positions identified in Table 1 are shown. As in Table 1, the C-termini are omitted from this analysis. The sequences of human βIII- and βV-tubulins are included for easier comparison.*

*^a^Species and accession numbers. Human = Homo sapiensβVI (NP_110400); Mouse = Mus musculus βVI (NP_001074440); Chicken = Gallus gallus βVI (NP_990776); Duck = Anas platyrhynchus (mallard) βVI (XP_005010451): Gecko = Gekko japonicus (Schlegel’s Japanese gecko) (XP_015268461); Turtle = Chelonia mydas (green sea turtle) (XP_007059846). Xenopus = Xenopus laevis (African clawed frog) (XP_031750537); Channa = Channa argus (northern snakehead fish) (KAF3690084); Molly = Poecilia Formosa (Amazon molly) (XP_007548412); Cod = Gadus morhua (Atlantic cod) (XP_030204426).*

There is probably some significance to these differences. Mammalian erythrocytes do not contain microtubules, whereas those of birds and reptiles do (Behnke, 1970a,b). Certainly, erythrocytes of animals exposed to the atmosphere would have high concentrations of oxygen, so it is not surprising that non-mammalian βVI contains the cysteine cluster while mammalian βVI does not. Significantly, slightly higher concentrations of glutathione have been observed in duck erythrocytes than occur in human erythrocytes and much higher concentrations of superoxide in zebra finch erythrocytes than in mouse erythrocytes (Dimant et al., 1955; Stier et al., 2013). It appears, therefore, that of the vertebrate βVI isotypes, only avian and some reptilian βVI have the cysteine cluster (Table 2). The fact that chicken βVI contains more of the key residues than does human βVI raises the possibility that these residues may help the cysteine cluster do its job and that the ones most relevant to dynamicity may be the others; these residues may be involved in strengthening, weakening, or altering the conformation near the cysteine cluster.

## The β-Tubulins of Cephalopod Mollusks

Among the animals, vertebrates have probably the most advanced and complex nervous systems (Green et al., 2015). One property of vertebrate brains is that they can grow new neurons, even in adulthood (Lindsey and Tropepe, 2006; Lemaire et al., 2012). Among the invertebrates, the cephalopod mollusks, specifically octopi and cuttlefish appear to be the most intelligent (Fiorito and Scotto, 1992; Godfrey-Smith, 2013). The cognitive abilities of cephalopods resemble those of vertebrates, as reviewed by Schnell and Clayton (2021). Interestingly, octopi can rejuvenate their brains (Di Cosmo et al., 2018). Some of these characteristics have led to the hypothesis that cephalopod mollusks and vertebrates are examples of convergent evolution, possibly arising from competition for similar ecological niches between these mollusks and fish (Packard, 1972; Kröger et al., 2011). The convergence has been noted in the structures of the cephalopod and vertebrate brains (Hochner et al., 2003; Moroz, 2009; Vitti, 2013; Shigeno and Ragsdale, 2015). Shigeno et al. (2018), summarizing a great deal of work in the area, describe the neural systems of cephalopods as “showing similarities to the cerebral cortex, thalamus, basal ganglia, midbrain, cerebellum, hypothalamus, brain stem, and spinal cord of vertebrates.” More specifically, the ratio of brain weight to total body weight in some cephalopods is higher than that of fish and lower than that of mammals and the vertical lobe of the octopus brain is similar to the hippocampus of mammals (Packard, 1972). This convergence would have occurred long after the ancestors of mollusks and vertebrates diverged over 520 million years ago (Valentine et al., 1999).

The question I am asking here is this: if there has been convergent evolution between cephalopods and vertebrates in their nervous systems, manifested at the organ level, could it also manifest itself at the molecular level? As mentioned above, βIII plays an important role in neuronal regeneration in vertebrates. Since, octopi can remake their entire brains, it is likely that a tubulin similar to βIII would be useful for this purpose in octopi. Perhaps the high dynamicity of microtubules formed from αβIII could be relevant. In addition, the learning and memory area in octopus brain exhibits long-term potentiation mediated by glutamatergic neurons (Hochner et al., 2003). In vertebrates, glutamate induces oxidative stress in neurons by binding the NMDA receptor, which causes a significant increase in the intracellular concentration of reactive oxygen species, including nitric oxide (Li et al., 2001; Lipton, 2006; Raju et al., 2015). Glutamate also induces oxidative stress in cerebral vascular cells (Parfenova et al., 2006). Silencing βIII allows glutamate to kill differentiating neurons (Guo et al., 2010). Since cephalopod mollusks have glutamatergic neurons, it would not be surprising if they also require a protein analogous to βIII-tubulin to protect them from the reactive oxygen species produced by glutamate. It would be worth doing an analysis of reactive oxygen species in these cells, including superoxide and nitric oxide. Finding elevated levels of these would strengthen the argument that one role of the β-tubulins containing residues resembling the conserved residues in vertebrate βIII would be to provide protection from oxidative damage. If cephalopod neuronal cells could be cultured, it would be useful to examine their microtubule dynamics and to mutagenize some of the residues discussed here to see how the cells’ growth and survival are affected. Another consideration is that if these mollusks undertake a dramatic reorganization of their brains, that would probably necessitate something similar in their microtubules, for which something like βV, would be useful, since disrupting microtubules is a signature feature of βV (Bhattacharya and Cabral, 2004; Salinas et al., 2014).

Table 3 shows that two octopi, *Enteroctopus dofleini* and *Octopus bimaculoides*, have the cysteine cluster found in βIII. Overall, they share seven positions where βIII differs from the other isotypes and also share four positions with both βIII and βV. The cuttlefish *Sepia officinalis* has two isotypes of β-tubulin, one of which has the cysteine cluster and which share 4 or 5 residues with βIII. A desirable experiment would be to see if the *Sepia* isotype with the cysteine cluster is associated with neurons. The β-tubulin of the squid *Doryteuthis pealeii*, which does not remake its own brain, does not have the cysteine cluster and does not share many residues with βIII. It appears that having a cysteine cluster may be required for neuronal regeneration in vertebrates and in cephalopod mollusks and that certain other residues in β-tubulin may be important for this function as well.

**TABLE 3:** Similarities in Structure to βIII and βV in Non-vertebrate β-Tubulins.

#	Human βIII						Non-cephalopod					Other β-tubulins						
		Cephalopod β-tubulins					Mollusc β-tubulins					Invertebrates			Other Eukaryotes			
		So1	So2	Ed	Ob	Do	Ac	Cg	Ls1	Ls2	Hd	Dm1	Dm2	Em	Nc	At	Gi	Tp
33	S	T	T	**S**	**S**	T	T	T	T	T	T	T	G	M	**S**	T	**S**	T
35	N	S	T	V	V	T	T	T	T	**N**	T	A	S	S	V	Q	E	T
37	V	H	H	Q	Q	H	H	H	H	H	H	R	L	N	N	**V**	R	H
48	S	N	**S**	Y	Y	N	T	N	N	N	N	N	E	N	N	D	N	N
55	S	T	T	**S**	**S**	T	T	T	**S**	**S**	T	**S**	**S**	Q	**S**	**S**	A	T
56	S	G	G	**S**	**S**	G	G	G	G	G	G	G	G	G	G	G	G	G
57	H	G	G	G	G	G	G	G	G	G	G	G	G	G	N	G	G	G
80	A	P	P	P	P	P	P	P	P	**A**	P	P	P	P	P	P	P	P
83	H	Q	Q	Q	N	Q	Q	Q	Q	Q	Q	Q	Q	K	Q	Q	Q	Q
84	L	**L**	I	**L**	**L**	I	I	I	I	**L**	I	**L**	**L**	**L**	**L**	I	I	**L**
91	I	V	V	V	V	V	V	V	V	V	V	V	V	**I**	V	V	V	V
124	C	A	**C**	**C**	**C**	A	S	A	A	S	A	A	**C**	**C**	A	A	S	A
126	N	S	**N**	G	G	S	**N**	S	**N**	**N**	S	S	**N**	A	G	**N**	A	G
155	V	I	I	I	I	I	M	I	M	M	I	I	I	I	I	I	I	**V**
189	L	I	V	V	V	V	V	V	V	V	V	V	I	V	V	V	V	V
218	A	T	T	T	P	T	T	T	T	S	T	T	S	P	S	**A**	T	T
239	S	C	C	C	C	C	C	C	C	**S**	C	C	C	**S**	**S**	C	C	C
275	A	S	S	S	S	S	S	S	S	S	S	S	S	S	S	S	S	S
315	T	**T**	A	A	A	A	A	A	A	A	A	A	A	A	A	A	A	A
332	A	N	N	S	S	N	N	N	N	N	N	N	**A**	E	N	N	N	N
333	I	**I**	V	**I**	**I**	V	V	V	V	V	V	**I**	V	T	V	**I**	**I**	V
335	S	**S**	N	N	N	N	N	N	N	N	N	N	N	T	N	N	N	N
351	V	T	T	T	T	T	T	T	Q	T	T	T	T	T	T	**V**	V	S
364	S	**S**	**S**	**S**	**S**	**S**	**S**	**S**	**S**	**S**	**S**	**S**	**S**	A	**S**	A	A	A
365	S	A	A	A	A	A	A	A	A	A	**S**	A	**S**	G	**S**	**S**	A	V

*Residues in the non-vertebrate β-tubulins that are the same as their equivalents in human βIII are indicated in **BOLD**. Key: So1 = Sepia officinalis (common cuttlefish) Isotype 1 (CCG28034) So2 = Sepia officinalis Isotype 2. (CCG28035). En = Enteroctopus dofleini (Giant Pacific octopus) (AAA16611). Ob = Octopus bimaculoides (California two-spot octopus) (KOF91916). Do = Doryteuthis pealeii (longfin inshore squid) (AAU11524). Ac = Aplysia californica (California sea hare) (NP_001191530). Cg = Crassostrea gigas (Pacific oyster) (BAD80737). Ls1 = Lymnaea stagnalis (great pond snail) Isotype 1 (AOV18890). Ls2 = Lymnaea stagnalis Isotype 2 (AOV18892). Hd = Haliotis diversicolor (abalone) (AEW42984). Dm1 = Drosophila melanogaster at 56D, isoform B (NP_523795). Dm2 = Drosophila melanogaster at 60D, Isoform (NP_001286835). Em = Echinococcus multilocularis (tapeworm) (CDI98193). Nc = Neurospora crassa (red bread mold) (AAA33617) At = Arabidopsis thaliana (thale cress) (AAA32757). Gi = Giardia lamblia (XP_001707388). Tp = Tetrahymena pyriformis (CAA31258)*

One question that is important to address is the possibility that the residues of interest play roles in protecting the microtubules of cephalopod mollusks from temperature extremes. Detrich et al. (2000) examined Antarctic fish, which are highly cold-adapted, and identified positions 202, 280, and 285 in β-tubulin as playing roles in stabilizing microtubules against temperature extremes (in the numbering used here, these would correspond to positions 200, 278, and 283). These are not the same as, nor adjacent to, the residues we have been studying here. However, that is not to say that they do not play a functional role. Intra-molecular interactions could very well cause the residues we are examining to influence other residues and increase the stability of the tubulin molecule. A closer study of the interactions among residues in the interior of the tubulin dimer could be a useful complement to studies of the residues involved in interactions between tubulin molecules.

## β-Tubulins From Other Invertebrates

Looking at the β-tubulin sequences of other mollusks, we can see that none of them has the cysteine cluster, but that one of them, the great pond snail *Lymnaea stagnalis*, has S239. Re-emphasizing that C239 is almost universally present in animals, plants and protists (with the exception of vertebrate βIII, βV, and βVI), it is surprising to see the exception in *Lymnaea*. This organism actually has two β-tubulin isotypes, neither of which has the cysteine cluster, and only one has S239. It is interesting that *Lymnaea* has a rudimentary lung (Dillon, 2006). The tissue distributions of the two *Lymnaea* β-tubulin isotypes are not known, but would be worth exploring; it is conceivable that having S239 in one of them may protect the microtubules from reactive oxygen species.

Vertebrates and cephalopod mollusks are not the only animals that exhibit adult neurogenesis; this phenomenon has been observed in insects, including the cricket and *Drosophila* (Scotto-Lomassese et al., 2002; Li and Hidalgo, 2020). Table 4 shows that, of two *Drosophila* β-tubulin isotypes, one has the cysteine cluster and shares several residues with βIII. Interestingly, this particular isotype (Tub60D) occurs in sensory neurons and in the cap cells of chordotonal organs, which may function in organizing the nervous system; this particular isotype is not required for formation of neurons but could play a role in their function. It may also play a role in making the microtubule arrays in the cap cells less rigid (Dettman et al., 2001). In this latter respect, it is interesting that mammalian βIII, when mixed with other β-tubulin isotypes, makes very dynamic microtubules, which could lead to overall less rigidity (Panda et al., 1994; Vemu et al., 2016). It is intriguing that while the other *Drosophila* β-tubulin isotypes have some resemblance to the other animal β-tubulin isotypes, Tub60D is considered more divergent (Hoyle and Raff, 1990), and yet, in its divergence, it has acquired some resemblance to vertebrate βIII–tubulin, consistent with the argument of this being a kind of convergent evolution. It is perhaps relevant that *Drosophila* brains are subject to high oxidative stress (Haddadi et al., 2014). Also, adult neural regeneration occurs in Platyhelminthes, such as the tapeworm *Echinococcus* (Olson et al., 2018). This too has the cysteine cluster. Tapeworms infect the guts of their hosts and are subjected to a wave of reactive oxygen species produced by the host (Secombes and Chappell, 1996; Hammerschmidt and Kurtz, 2009). It is thought that tapeworms evolved mechanisms of penetrating other tissues of the host in order to escape these factors (Read and Skorping, 1995; Mulcahy et al., 2005). If the cysteine cluster plays a role in protecting the microtubules against reactive oxygen species, it is not surprising that it would be present in tapeworm tubulin.

**TABLE 4:** C-Terminal Regions of Various β-Tubulins.

Vertebrate βIII-tubulins	
*Homo*:	YQDATAEEEGEMYEDDEEESEAQGPK
*Mus*:	YQDATAEEEGEMYEDDDEESEAQGPK
*Gallus*:	YQDATAEEEGEMYEDDEEESEAQGAK
*Gekko*:	YQDATAEEEGEMYEDDEEESEAQGAK
*Xenopus*:	YQDATAEEEGEMYEDDEEESEAQGK
*Salmo*:	YQDATTEEEGEMYEDDEEESESQAR
**Other Human β-tubulin isotypes**	
βI	YQDATAEEEEDFGEEAEEEA
βIIA	YQDATADEQGEFEEEEGEDEA
βIIB	YQDATADEQGEFEEEEGEDEA
βIVA	YQDATAEEGEFEEEAEEEVA
βIVB	YQDATAEEEGEFEEGAEEEVA
βV	YQDATANDGEEAFEDEEEEIDG
βVI	FQDAKAVLEEDEEVTEEAEMEPEDKGH
**Cephalopod mollusc β-tubulin isotypes**	
*Sepia officinalis* Isotype 1	YQDATAEEEAEMDEEEEDVA
*Sepia officinalis* Isotype 2	YQDATTEEEILIEEAEDEEA
*Enteroctopus dofleini*	YQEARSTDSDEYDNEEYYNQQEE
*Octopus bimaculoides*	YQEARATDSDEYDDEDQYNEQE
*Doryteuthis pealii*	YQDATAEEEGEFEEEGEEDA
**Other mollusk β-tubulins**	
*Aplysia californica*	YQDATAEDEGEFDEEEGDEGGEEYA
*Crassostrea gigas*	YQDATAEEEGEFEEEEGEEEAQ
*Lymnaea stagnalis* Isotype 1	YQDATAEDEGEFDEEEAEGEGQEYA
*Lymnaea stagnalis* Isotype 2	YQEATIDEDVEVEEGADEDAGDL
*Haliotis diversicolor*	YQDATAEEEGEFDEEEGEADEA
**Other invertebrate β-tubulins**	
*D. melanogaster* (56D, Isoform B)	YQEATADEDAEFEEEQEAEVDEN
*D. melanogaster* (60D, Isoform B)	LVSEYQQYQEATADDEFDPEVNQEEVEGDCI
*Nasonia vitripennis*	YQEATTEEDFETEDAGDDFETCDQE
*Bombyx mori*	YQEATAEDDTEFDQEDLEELAQDEHHD
*Echinococcus multilocularis*	EYQQYQEVGIDDDYGEEEAAPEE
**Other eukaryotic β-tubulins**	
*Neurospora crassa*	YQDAGVDEEEEEYEEEAPLEGEE
*Arabidopsis thaliana*	YQDATAGEEEYEEEEEEYET
*Giardia lamblia*	YQEAGVDEGEEFEEEEDFGDEYA
*Tetrahymena pyriformis*	YQDATAEEEGEFEEEEGEN

*Note that the vertebrate βIII-tubulins end with a basic amino acid and all have a tyrosine and a serine close to their C-termini; these features are absent in the other human β-tubulins. The serine near the C-terminus of βIII is phosphorylated (Khan and Ludueña, 1996; Ludueña et al., 1998). These features are absent from the C-termini of the non-vertebrate β-tubulins.*

## Dissecting the βIII-Tubulin Isotype and Its Roles in Neurons

It is clear from the literature that βIII plays an important role in neurons, especially in neurogenesis. This role could involve both the protection of microtubule assembly from reactive oxygen species and the high dynamicity of microtubules containing the αβIII dimer. Here I will evaluate specific regions of βIII and speculate as to what they might be doing and how to test these speculations.

### The Cysteine Cluster 124–129 (CENCDC)

It is clear that this region is important in the function of βIII because every βIII in vertebrates contains it. It has the same topography of cysteines as does von Willebrand’s factor and could very well interact with glutathione or engage in a sulfhydryl-disulfide interchange. Every tissue in which βIII is most strongly expressed is also rich in reactive oxygen species. A similar argument may apply to βV, although the actual sequence of the cluster is CEHCDC, slightly different from that of βIII. βV is found in high concentrations in airways (Nakamura et al., 2004). The fact that it differs from βIII in having a histidine at position 126 instead of an asparagine may mean that the putative protection against reactive oxygen species may not be so stringent for βV or that the cysteine cluster in βIII may have an additional function. The cysteine cluster in the form CENCDC occurs in one of the *Drosophila* β-tubulin isotypes as well as in the cuttlefish *Sepia*. It is slightly different in *Enteroctopus* and *Octopus*, where it is CEGCEC, and the tapeworm *Echinococcus*, where it is CEACGC.

It is probably significant that most of the βVI isotypes lack the cysteine cluster, the exceptions being CESCDC in the chicken and CENCDC in the Atlantic cod. The βVI isotype occurs in the microtubules of platelets and erythrocytes; one would expect erythrocytes to be rich in oxygen. Since, mammals lack microtubules in their erythrocytes, the requirement for a cysteine cluster in mammalian βVI would be less stringent, if one accepts the argument outlined above. All the non-mammalian vertebrates have microtubules in their erythrocytes. One could argue that birds, who breathe substantial amounts of oxygen while flying, may also profit from the cysteine cluster in their erythrocyte microtubules. This argument may not apply so strongly to reptiles, amphibians and fish, so it is not surprising that most of them lack the cysteine cluster in βVI. What purpose the cluster might serve in the Atlantic cod is not clear.

There are several experiments that could illuminate the role of the cysteine cluster. One would be to prepare a mutant of βIII with a serine at position 124 instead of a cysteine. This mutant could be included in a dimer with α and its properties examined. The dynamicity of microtubules formed from this dimer should be determined and the sensitivity to reactive oxygen species could be examined as well as its interaction with glutathione. If the C124S mutant were to have normal dynamics but be very sensitive to reactive oxygen species such as superoxide, then we may conclude that the role of the cysteine cluster may be to protect microtubule assembly from oxidation. If microtubule dynamics were affected that would imply a role in influencing the conformation of the tubulin molecule to permit this; I shall discuss this possibility further below. Also, βIII mutagenized with C124S could replace wild-type βIII in developing neurons and we could learn about the specific role of that residue in regulating neurogenesis.

It is past time for βV to be either purified as an αβV dimer or else synthesized, dimerized with α, and its properties examined *in vitro*. An isotype-specific monoclonal antibody could be useful in purifying βV, particularly because it would allow identification of its post-translational modifications. Measurement of its dynamic and drug-binding properties would illuminate its role *in vivo* as well as that of βIII. Because it is sometimes over-expressed in cancer cells, essentially taking the place of βIII, it could have properties similar to those of βIII. Any differences could be of great interest and allow further hypotheses about the role of βIII in neurons, where βV Is expressed only in limited quantities.

### S239

As mentioned above, this is an unusual feature of βIII, since virtually all animals, plants and protists express C239. However, every vertebrate βIII, βV, and βVI appears to have S239 instead of C239. We know that C239 is uniquely susceptible to alkylating agents (Bai et al., 1989). It is therefore reasonable to speculate that the tissues and organisms whose β-tubulin contains S239 are exposed to high concentrations of reactive oxygen species. This could be tested using the βIII mutant S239C in an αβIII dimer and measuring susceptibility of its assembly to inhibition by physiologically significant agents such as superoxide, nitric oxide, hydrogen peroxide, and peroxynitrite. Replacement of wild-type βIII by this mutant in developing neurons could demonstrate whether this residue plays a role in neurogenesis. Similarly, neurons grown in culture expressing the mutant could be subjected to oxidative stress by treatment with glutamate and their survival compared with that of neurons expressing wild-type βIII.

Glutamate causes production of nitric oxide in mouse neurons and specifically nitrosylates a large number of proteins including tubulin (Raju et al., 2015). The nitrosylated residues include C354 in βIIa, βIIc, βIII, and βV as well as C239 in βIIc. C354 is almost universally conserved in eukaryotic β-tubulins and is easily cross-linked to C239 (Little and Ludueña, 1985). The fact that C239 is only nitrosylated in βIIc and not in the other β isotypes in which it occurs, suggests that the role of C239, and perhaps also S239, may be more complex than we have imagined.

### C-Termini

The C-termini of both α- and β-tubulin have been the subject of many studies. Due to their high negative charges, they are very likely to be on the surface of the tubulin molecule, both when it is a free dimer and also when it is part of a microtubule. Not only is the encoded sequence rich in negatively charged residues, but this part of the tubulin molecule is often polyglutamylated, which makes it even more negatively charged (Janke and Magiera, 2020). The C-terminal region is necessary for a microtubule to form. Removal of the last 21 residues of the C-terminal region of βIII leaving the new C-terminus as YQDAT, had very little effect on its ability to assemble, while removal of one more residue diminished assembly by about 75% and removal of still one more residue abolished assembly entirely (Joe et al., 2009). Interestingly, ability to assemble could be fully restored in a chimeric mutant where the C-terminal region of βI replaced that of βIII. The converse was also true (Joe et al., 2009). This suggests that, at least for evaluating the role of the C-terminal domain on assembly, the specific residues did not matter so much as the presence of a region with similar overall properties.

This is consistent with the sequences of the various vertebrate βIII isotypes. The immediate impression that comes to mind when one compares the C-terminal regions of vertebrate βIII isotypes is that they are very variable in length (Table 4). One could argue that, in contrast to the rest of the protein, in analyzing the possible function of the C-terminal domain of βIII, it may be more productive to look at overall characteristics of the C-terminal domain rather than compare residues at specific positions. Four features unique to βIII are apparent. First, there is a methionine present at the same position (436) in each vertebrate βIII; this is absent from every other vertebrate β-tubulin isotype, and the role of this methionine has never been investigated. The methionine is followed by a tyrosine, again present in every βIII and absent from the other β-tubulin isotypes. The methionine is not present in the C-termini of the invertebrate β-tubulins discussed here.

The next site of interest is S444, which can be phosphorylated (Ludueña et al., 1998). Phosphorylation of this residue promotes binding to microtubule-associated proteins (Khan and Ludueña, 1996), which would be useful for βIII, because these proteins are abundant in the brain.

Finally and most strikingly, the C-terminal residue of βIII always appears to be a basic residue, either lysine or arginine. None of the other vertebrate β-tubulin isotypes share this feature, nor does it occur in the invertebrate tubulins that have been discussed here. One could perhaps speculate that this residue could form an electrostatic bond with a negatively charged residue in the C-terminus of an adjacent tubulin molecule on the microtubule and could perhaps play a role in the high stability of microtubules made of purified αβIII (Vemu et al., 2016). If this C-terminal lysine or arginine forms an electrostatic bond with a negatively charged residue in the same molecule that could act as a powerful stabilizer of the tubulin, making it more difficult to bind to drugs.

Whatever the role of the C-terminal, it is difficult to conclude that it is involved in neuronal regeneration since the C-terminal regions of the cephalopod mollusks that can remake their own brains do not resemble those of vertebrate βIII. The same is true for the other invertebrate β-tubulins discussed here.

The possible roles of the C-terminal residues would not be that difficult to explore. The residues discussed above could be mutated and the properties of the corresponding αβIII dimers explored as described above. Residues to mutate would include tyrosine 437 and serine 444. Removal of the C-terminal lysine would also be logical. The parameters to be measured would include dynamicity of the microtubules they form as well as the stability of the tubulin dimer. The high negativity of the C-terminus could contribute to the electrical properties of the microtubules (Tuszynski et al., 1995) and the effect of these mutations on that would be useful to examine.

### The Conserved Residues Mutated in Cancer Patients

Perhaps the most interesting regions of βIII to examine are not necessarily discrete entities but rather sets of residues that were found to be mutated in breast cancer patients with high grade tumors (Wang et al., 2017). Only one of these involved a single mutation (A275T in βIII). Most of these involved a set of several mutations. In some cases, residues in βIVB were mutated to the corresponding residues in βIII. Thus, one patient had mutations at positions 124, 126, 155, 189, 218, and 239; another patient had mutations at positions 275, 315, 332, 333, 335, 351, and 365; a third patient had mutations at positions 332, 333, 335, 351, and 365. The fact that these mutations were accompanied by aggressive high-grade cancers suggests that these positions are critical for the correct dynamics of tubulin, but these results do not allow us to single out one particular residue. The group of residues in βIII that appear frequently in this set are thus C124, N126, V155, I189, A218, S239, A275, T315, A332, I333, S335, V351, S364, and S365. These observations suggest that these particular residues may have significant conformational effects on the tubulin molecule, either stabilizing the conformation or making it more flexible. Making mutants of βIII in which these residues are mutated to the corresponding residues in other β-tubulin isotypes may result in identifying the conformational roles they may play. Differential scanning calorimetry may be a good tool for this study (Schwarz et al., 1998). It is interesting hat this set of residues overlaps with some of the other points of interest. Specifically, we have the mutations at positions 124 and 126 in the cysteine cluster. I have already hypothesized that the cysteine cluster could protect microtubule assembly from reactive oxygen species. That is certainly consistent with generating an A124C mutant in βIVB. However, it may not explain a possible effect of an S126N mutant in βIVB. Those two mutations occurred in the same patient, so one need not conclude that both had functional significance. However, we have shown that the C124S mutant of βIII binds colchicine much less well than does wild-type βIII (Joe et al., 2008). Since position 124 is not part of the colchicine-binding site, it is likely that we are looking at a conformational effect rather than an effect involving a sulfhydryl group interaction. Similarly, the S126N mutation, which is not likely to affect the anti-oxidant function of the cysteine cluster, could very well alter the conformational properties of tubulin. Along the same lines, the βV isotype is known to disrupt microtubules, which implies a unique conformational role for βV (Bhattacharya and Cabral, 2004). However, Bhattacharya and Cabral (2009) observed that the S239C mutant of βV lacks the microtubule disrupting ability; cumulatively, this evidence indicates that position 239 may not just be involved in protecting microtubules from oxidation but also in regulating tubulin conformation. It is also interesting that every mutation of C239 to residues other than serine (leucine, tyrosine, arginine, and proline) were associated only with low-grade cancers while the C239S mutation was associated with high-grade cancers. None of these mutants would be able to be react well with reactive oxygen species. This raises the possibility that this position also may be associated with regulation of the tubulin conformation.

Finally, we should look at the invertebrates that have the same residues as vertebrate βIII in positions that are highly conserved positions in vertebrate βIII (Table 5). These are also the ones that have either the cysteine cluster (*Enteroctopus*, *Octopus*, *Drosophila, Nasonia, Bombyx*) or S239 (*Lymnaea, Echinococcus*). This raises the possibility that the functions of the cysteine cluster and of S239, whatever they may be, are influenced by the nature of the residues that occupy these other positions. For the purposes of this table, I have presented the data from βIII in two sets. The first set, called “HMBG βIII,” is based on the sequences of βIII from humans (*Homo)*, mice (*Mus*), cows (*Bos*), and chickens (*Gallus*). βIII-Tubulin from each of these is identical at the positions of interest and are, in a sense, being privileged because they may be the only organisms in which the functional properties of their βIII has been examined (Lopata and Cleveland, 1987; Banerjee et al., 1992; Panda et al., 1994; Joe et al., 2008; Guo et al., 2010, 2011, 2018; Vemu et al., 2016). The second set, called “Vert βIII” refers to the 8 residues that appear to be conserved in all vertebrate βIII-tubulins, as described above.

**TABLE 5:** Number of Positions That Are Conserved in Vertebrate βIII or Both βIII and βV that Are Also Conserved in Non-Vertebrate β-Tubulins.

	# in HMBG βIII	# in HMBG βIII AND βV	# in Vert βIII	Cysteine “cluster” 124-129	Region around Pos’n 239
**Organism/Isotype)**					
**Cephalopod Molluscs**					
*Sepia* Isotype 1	5	3	1	No (AES**C**D**C**)	TT**C**L
*Sepia* Isotype 2	4	1	3	Yes (**C**EN**C**D**C**)	TT**C**L
*Enteroctopus*	7	4	3	Yes (**C**EG**C**E**C**)	TT**C**L
*Octopus*	7	4	3	Yes (**C**EG**C**E**C**)	TT**C**L
*Doryteuthis*	1	0	0	No (AES**C**D**C**)	TT**C**L
**Other Molluscs**					
*Aplysia*	2	0	0	No (SEN**C**D**C**)	TT**C**L
*Crassostrea*	1	0	0	No (AES**C**D**C**)	TT**C**L
*Lymnaea* Isotype 1	3	1	0	No (AEN**C**D**C**)	TT**C**L
*Lymnaea* Isotype 2	7	2	2	No (SEN**C**D**C**)	TTSL
*Haliotis*	2	1	0	No (AES**C**D**C**)	TT**C**L
**Other Organisms**					
*Drosophila* (Dm1)	4	2	1	No (AES**C**D**C**)	TT**C**L
*Drosophila* (Dm2)	7	4	2	Yes (**C**EN**C**D**C**)	TT**C**L
*Nasonia*	8	3	2	Yes (**C**EN**C**D**C**)	TT**C**L
*Bombyx*	10	4	3	Yes (**C**EN**C**D**C**)	TT**C**L
*Echinococcus*	4	3	3	Yes (**C**EA**C**D**C**)	TTSL
*Neurospora*	6	3	2	No (AEG**C**D**C**)	TVSL
*Arabidopsis*	7	3	2	No (AENSD**C**)	T**CC**L
*Giardia*	3	2	1	No (SEA**C**D**C**)	TS**C**L
*Tetrahymena*	2	0	1	No (AEG**C**D**C**)	T**CC**L
**Human β-tubulins (for comparison)**					
βI (BAB63321)	1	0	0	No (AES**C**D**C**)	TT**C**L
βIIA (NP_001060)	1	1	0	No (SES**C**D**C**)	TT**C**L
βIIB (NP_821080)	1	0	0	No (SES**C**D**C**)	TT**C**L
βIII (AAL28094)	25	12	8	Yes (**C**EN**C**D**C**)	TTSL
βIVA (NP_001276058)	1	1	0	No (AES**C**D**C**)	TT**C**L
βIVB (NP_06079)	0	0	0	No (AES**C**D**C**)	TT**C**L
βV (Q9BUF5)	12	12	4	Yes (**C**EH**C**D**C**)	TTSL
βVI (NP_110400)	6	2	5	No (SES**C**D**C**)	TTSL
Chicken VI	9	4	7	Yes (**C**ES**C**D**C**)	TTSL

*This table highlights the two features that may address oxygen toxicity: the “cysteine cluster” (C124, C127, C129) and S239. Since human βIII is obviously identical to itself, it I only present to show the sequence of its cysteine cluster. Note that invertebrates that have one of these features tend to have more of the residues that have been highly conserved in the evolution of βIII in vertebrates. This pattern is not so obvious in plants, fungi and protists. The “cysteine cluster” and the region of position 239 in human β-tubulin isotypes and chicken βVI are included for purposes of comparison. Cysteine residues are emphasized in **BOLD**.*

The experiments to address this point would be to duplicate some of the mutations of βIII occurring in cancer patients and see if these result in alterations in the stability of the αβIII dimer or the dynamicity of microtubules containing this dimer.

The ultimate outcome of these experiments would be a better understanding of the role that tubulin conformation plays in microtubule dynamicity and drug-binding and gain us a better understanding of the role of βIII, which is most concentrated in neurons, in neuronal development. Similarly, we may be able to learn how tubulin functions in cephalopod neurons.

There is another potential bonus in studying the role of these residues. Banerjee et al. (1994, 1997) showed that a major reason that the αβIII dimer binds less well to colchicine analogs than do the αβII and αβIV dimers is that the conformational change induced in the tubulin molecule by the binding of these drugs is much slower in αβIII than in the other two dimers. Although this has not been directly tested, it is reasonable to suppose that the same would be true for the lesser effects of taxol and vinblastine on αβIII (Derry et al., 1997; Khan and Ludueña, 2003). In other words, even if a drug is designed to fit well into its binding site on αβIII, it may not be sufficient unless a way is found to cause the regions around these other residues to become more flexible. One could perhaps imagine designing a novel drug that fits better into its binding site on βIII and either doing further modifications of this drug or designing a second drug that would interact with some of the other residues in βIII that have roles in maintaining the conformation of βIII so as to allow the primary drug to fit better into its binding site. In short, the studies proposed here may allow the design of novel and improved anti-tumor drugs.

## Data Availability Statement

The datasets presented in this study can be found in online repositories. The names of the repository/repositories and accession number(s) can be found in the article/supplementary material.

## Ethics Statement

The studies involving human participants were reviewed and approved by Alberta Cancer Research Biobank. This manuscript only uses human subjects data that was previously approved for an earlier published study. The patients/participants provided their written informed consent to participate in this study.

## Author Contributions

The author confirms being the sole contributor of this work and has approved it for publication.

## Conflict of Interest

RL owns shares in OncoVista Innovative Therapies (San Antonio, TX, United States) and receives royalties from Santa Cruz Biotechnology (Dallas, TX, United States) for the sale of monoclonal antibodies to tubulin isotypes.

## Publisher’s Note

All claims expressed in this article are solely those of the authors and do not necessarily represent those of their affiliated organizations, or those of the publisher, the editors and the reviewers. Any product that may be evaluated in this article, or claim that may be made by its manufacturer, is not guaranteed or endorsed by the publisher.
